# Acupressure for agitation in nursing home residents with dementia: study protocol for a randomized controlled trial

**DOI:** 10.1186/1745-6215-15-410

**Published:** 2014-10-27

**Authors:** Rick Yiu Cho Kwan, Mason Chin Pang Leung, Claudia Kam Yuk Lai

**Affiliations:** School of Nursing, The Hong Kong Polytechnic University, Hong Kong, China; Department of Rehabilitation Sciences, The Hong Kong Polytechnic University, Hong Kong, China

**Keywords:** Acupressure, Dementia, Nursing home, Salivary cortisol, Agitation

## Abstract

**Background:**

Agitation is prevalent among people with dementia (PWD) in nursing homes. It frustrates both the PWD and their caregivers. Acupressure is a non-pharmacological intervention whose effectiveness is supported by preliminary studies. However, there is still a dearth of evidence to explain its effect for clinical use and further research. The present study is being conducted primarily to investigate the effects of acupressure as compared with sham-acupressure and usual care.

**Methods/design:**

This study is a multicenter, assessor/participant/statistician-blinded, parallel group, randomized controlled trial taking place in Hong Kong nursing homes. We have been recruiting PWD over 65 years of age in nursing homes, who are experiencing agitation; 99 participants will be recruited in order to demonstrate a significant effect difference (that is, f =0.27) with a power of 0.8 and a significance level of 0.05 among the three groups. Participants are assigned by permuted block randomization into three groups in a 1:1:1 ratio. In the acupressure group, participants receive acupressure at the Fengchi (GB20), Baihui (GV20), Shenmen (HT7), Niguan (PC6) and Yingtang (EX-HN3) acupoints. In the sham-acupressure group, participants receive pressure on five non-acupoints. In the usual-care group, participants receive no intervention apart from the care provided by the nursing homes. Participants assigned to the sham-acupressure and usual-care groups receive free acupressure, like those in the acupressure group, after completion of the study. The whole study lasts for 30 weeks, and its primary outcome measure is agitation. The general estimated equation model will be used to compare the effects among groups and time points. The trial is currently recruiting participants.

**Discussion:**

This trial will provide a higher quality of evidence than previous studies on the use of acupressure for agitation in PWD. It will also provide newer evidence on acupressure in the population of PWD with agitation for clinical application and further research, including the effect on moderating stress, the delayed effect, the added effect on the placebo, and the effect on moderating the participant’s use of psychotropic drugs.

**Trial registration:**

Centre for Clinical Trials Clinical Trials Registry: CUHK_CCT00347 (Registration date: 13 December 2012).

## Background

Agitation is excessive, disruptive and inappropriate activities with verbal, vocal and motor components that are unexplained by apparent needs or confusion [1–3]. It can also be conceptualized as one of many behavioral or psychological symptoms of dementia [4] or as a neuropsychiatric symptom [5]. Agitation is the term most commonly used by professionals in long-term care facilities to describe dementia-related behavioral symptoms [6, 7]. Agitation is often the most prevalent neuropsychiatric symptom in residential care homes (RCHs), with prevalence ranging from 20.5% to 63% [8–11]. Agitation decreases quality of life [12, 13], and causes many negative consequences, such as falling [14] and use of restraints [15]. Agitation in PWD also increases caregivers’ burden [16, 17] and distresses formal caregivers [17, 18].

Acupressure is a non-pharmacological intervention whereby pressure is applied to specific acupoints on the body to treat a wide range of conditions or promote individual well-being [19]. It is a multi-modal therapy whose working mechanism can be explained by many different theories [20]. For example, traditional Chinese medicine (TCM) explains that acupressure stimulates the acupoints to enhance the energy or Qi flowing along the meridian, achieving therapeutic outcomes by improving the functions of body systems or Zangfu in the process [21]. Bio-chemical studies explain that stimulation of the acupoints by pressure may cause complex neuro-hormonal responses [22]. One of these responses may involve the hypothalamic-pituitary-adrenocortical axis to counteract the overproduction of cortisol and cause a relaxation response [23]. Similar manipulative therapies, such as massage and therapeutic touch, were shown to be useful in lowering the patient’s stress and cortisol after treatment [24, 25].

Preliminary studies [26–28] have shown that acupressure can reduce agitation in PWD. However, given that the participants and the RCH staff providing the outcome observation were not blinded in the previous studies, the extent of the placebo effect could not be estimated. Previous studies showed that manipulative therapies, such as massage [23, 29] and therapeutic touch [25, 30], had also reduced the agitation and stress of PWD. These studies led the present researchers to hypothesize that acupressure may produce an agitation-reducing effect by mediating the stress hormone (that is, cortisol) through tactile stimulation alone, instead of via acupoint stimulation. Many other factors that might account for the effect were not reported in previous studies, such as the delayed effect of the intervention and the use of psychotropic drugs.

Therefore, we designed the present study primarily to investigate the effectiveness of acupressure for reducing agitation, compared with sham-acupressure and usual care. Meanwhile, we further investigated the effect of acupressure on stress moderation (that is, reduction of cortisol level), the delayed effect, the efficacy of acupressure in addition to the placebo effect (that is, compared with the sham-acupressure), and whether acupressure leads to a reduction in psychotropic drug use.

## Methods/design

### Design

This study is a multi-center, assessor/participant/statistician-blinded, parallel group, randomized controlled trial. Participants are randomly allocated to three groups in a 1:1:1 ratio: the acupressure group, the sham-acupressure group, and the usual-care group. Eligible participants will be recruited from nursing homes in Hong Kong.

### Trial process

In order to recruit a sufficient number of participants in multiple nursing homes and balance the manpower demands, the whole study will be completed through six study cycles. The periods of each cycle partially overlap in order to efficiently utilize the manpower and study venues. Each study cycle lasts for 10 weeks, including a 2-week baseline period, a 2-week intervention period, and a 6-week follow-up period (Table 1). The whole study period lasts for 30 weeks.

**Table 1:** **Trial process chart of each study cycle**

	Baseline	Treatment phase	Follow-up phase		
Tasks	Week -2	Week 0	Week 3	Week 5	Week 8
**Participants/next-of-kin**					
Informed consent	x				
Sign the proxy consent	x				
Obtain participant’s assent	x				
Access medical record	x				
Physical examination	x				
**Intervention**					
Acupressure group (n =33)		x			
**Control**					
Sham-acupressure group (n =33)		x			
Usual-care group (n =33)		x			
**Outcomes**					
Agitation (CMAI)		x	x	x	x
Stress (salivary cortisol)		x	x	x	x
**Monitoring**					
Compliance with the intervention		x			
Reasons for withdrawal		x	x	x	x
Adverse events/safety		x	x	x	x

*CMAI*, Cohen-Mansfield Agitation Inventory.

### Recruitment procedures

**Figure 1:**
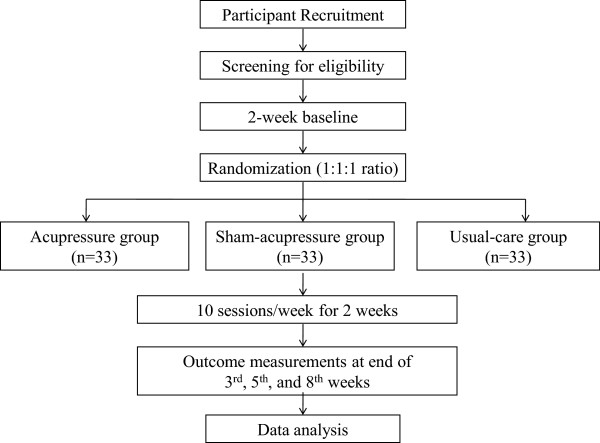
**Trial flow chart.**

We have invited all the RCHs in Hong Kong that are registered under the Social Welfare Department and fulfill the following criteria: (1) they have more than 100 beds; and (2) they provide beds for moderate/severe functioning elders (that is, Care and Attention Homes and Nursing Homes) [31]. The RCHs are invited by mail and followed up by telephone. Of the RCHs who were interested in participating, 12 RCHs were selected randomly to launch this study. Only 12 were selected because it was estimated from the pilot study [32] that at least 12 RCHs with more than 100 beds each would provide sufficient participants for the study (that is, 99 participants). Within the 12 selected RCHs, our research team is working closely with RCH staff to screen all the residents against the selection criteria. After screening, all eligible residents are invited to participate in the study by telephone. Participants are included only if they meet the selection criteria and provide assent/informed consent, and if the next-of-kin provide written informed proxy consent (Figure 1).

### Randomization

Permuted block randomization is used. The permuted block randomization list was generated by the web-based generator at http://www.randomization.com [33]. To guarantee allocation concealment, randomization is done by an independent research assistant who does not participate in any other parts of the research. Throughout the study period, the independent research assistant generates the randomization list and keeps the list. Information on the recruited participant (for example, name, age, and gender) is sent to the independent research assistant by email after the recruitment. The independent research assistant assigns the group code to each participant according to the randomization list and sends the information back to the researcher by email. The independent research assistant is blinded to the meaning of the group code.

### Blinding

Participants, next-of-kin, data collectors, nursing home staff (that is, the formal caregivers providing the Cohen-Mansfield Agitation Inventory (CMAI) data and the nurses administering the psychotropic drugs), physicians prescribing the psychotropic drugs, and the statistician will be blinded to the group allocation status; however, blinding is clearly not possible in the usual-care group. The group allocation status will only be revealed after completion of the study.

### Participants

This study focuses on older nursing home residents with dementia and agitation. Eligible participants should match all the inclusion criteria, and participants meeting any of the exclusion criteria should be excluded.

#### Inclusion criteria

Participants will be included if they fulfill the following criteria: (1) aged over 65 years, (2) documented as having dementia in the medical record, and (3) identified as having agitated behaviors for the period 1 month prior to the time of recruitment according to the criteria for agitation stated in the Instruction Manual for CMAI [34].

#### Exclusion criteria

Exclusion criteria are: (1) skin problems on acupoints (for example, skin breakdown, infection), (2) musculo-skeletal problems (for example, amputation of the acupoints), and (3) having received acupuncture/acupressure within the 8 weeks immediately prior to the day of recruitment.

### Intervention

This intervention protocol was developed according to the 2008 Medical Research Council guideline for developing complex interventions [35]. The intervention was developed based on three methods: a literature review, the Delphi process [36] and a pilot study. The literature review [37] identified articles discussing the theories possibly connecting the use of acupressure and the agitation. Evidence relevant to the use of acupressure on agitation in PWD was retrieved by a systematic review from the major databases. The aim of the literature review was to identify the existing evidence and develop theories to explain why acupressure might work. The relevant information retrieved from the literature was then summarized as a document for the subsequent Delphi process. In the Delphi process [37], a panel of six TCM experts specializing in acupuncture was consulted. All TCM experts on the panel were TCM practitioners registered in either Hong Kong or mainland China with a bachelors degree or above. The expert panel has rich experience in practicing TCM and clinical acupuncture, with a median of over 15 years in practice. The Delphi process first asked the panel experts to suggest the intervention components (that is, the selection of acupoints), the intervention dosage, and the rationales for the selection of components. They were then asked to give suggestions with reference to the information provided in the literature and their own professional knowledge and experience. Finally, consensus on the procedural details of the intervention protocol was reached through three cycles of stepwise anonymous discussion until the content validity index achieved 1.0. Although consensus on the intervention dosage (that is, the duration and frequency) was reached in the Delphi process, the narrative comments from the Delphi process showed that the dosage was based on the experts’ experience only because TCM theory does not discuss dosage. A pilot study was performed to provide supporting evidence for the dosage selection. In the pilot study [32], within the range of dosage suggestions given in the Delphi process, the effect of acupressure was compared among various dosages. It was found that shorter duration with higher frequency showed a larger effect. The dosage that showed the largest and most significant effect was used in the intervention protocol, which is to conduct the intervention twice a day for 2 weeks.

#### Acupressure group

In this group, we use five acupoints: Fengchi (GB20), Baihui (GV20), Shenmen (HT7), Niguan (PC6) and Yingtang (EX-HN3). The researchers first identify the acupoints; the acupoint identification method in this study is based on two textbooks [38, 39] with complete agreement from the expert panel. The researchers then apply pressure on the acupoints, following a light-strong-light pattern. They increase the pressure gradually until it reaches the optimal level, sustain the pressure at the optimal level for 3 minutes, gradually decrease it after 3 minutes and finish by kneading the pressed acupoints. The optimal pressure is defined by experiencing the Deqi sensation (that is, soreness, numbness, distention, heaviness) [40]. The optimal pressure to achieve the Deqi sensation differs among individuals. The sensation can be confirmed by asking the participants and observing their behavior (for example, frowning, withdrawing). When the pressure increases to a level where the Deqi sensation begins to be obviously felt by the participants or observed by the interventionist, the level of pressure is regarded as optimal.

Yintang (EX-HN3) is first pressed for 3 minutes. Baihui (GV20) and Fengchi (GB20) are then pressed simultaneously for another 3 minutes. Finally, Neiguan (PC6) and Shenmen (HT7) are pressed simultaneously for the last 3 minutes. For the bilateral acupoints (that is, GB20, PC6, HT7), the acupoints on one side are pressed in each session and they are pressed alternatively along sessions. Each acupressure session lasts for 9 minutes. Acupressure is performed twice a day, 5 days a week (from Monday to Friday) for 2 weeks. A course of acupressure consists of 20 sessions and lasts for 2 weeks.

#### Sham-acupressure group

In this group, five non-acupoints are used as controls. A non-acupoint is defined as a point on the body not falling on the acupoints depicted in the two TCM reference books [38, 39] and agreed by the members of the expert panel. The five non-acupoints are the root of the nasal bone (point 1), the olecranon of the ulna (point 2), the ulnar styloid process (point 3), the medial malleolus (point 4), and the head of the fibula (point 5). Pressure is applied on the five non-acupoints in the same way as in the AG. Point 1 is first pressed for 3 minutes. Points 2 and 3 are next pressed simultaneously for another 3 minutes. Finally, points 4 and 5 are pressed simultaneously for the last 3 minutes. For the bilateral points (that is, points 2, 3, 4, and 5), the points on one side are pressed in each session and they are pressed alternatively along sessions. The intervention dose and duration are the same as in the AG. The participants in this group receive a free course of acupressure sessions, identical to the one stipulated in the AG, after completing the study.

#### Usual-care group

In this group, participants receive no acupressure-related intervention. They receive a free course of acupressure sessions, identical to the one stipulated in the AG, after completing the study.

### Outcome measurement

The primary outcome in this study is the change of agitation (that is, CMAI score), and the secondary outcome is the change in stress ( measured by salivary cortisol concentration). The outcomes will be measured at the baseline (T0), the week immediately after completion of the intervention (T1), 2 weeks after completion of the intervention (T2), and 6 weeks after completion of the intervention (T3). The measurement at T1 aims to examine the immediate effect after the intervention, that at T2 aims to examine the peak effect, and that at T3 aims to examine the delayed effect. The decision to use the measurement interval of T2 and T3 was based on the result of the pilot study [32], where weekly repeated measurement showed that the effect of acupressure peaked at the time point 2 weeks after the completion of intervention and diminished to very close to baseline at the time point 6 weeks after the completion of intervention.

### Training and quality control

All research personnel, those conducting the intervention, and data collectors must attend an 8-hour training course consisting of one lecture and two skills workshops provided by two of the six members of the TCM expert panel. This training is intended to ensure that they comply with the research protocol. All personnel conducting the intervention have to pass a skill examination administered by one of the six members of the TCM expert panel. The test contents included correct identification of the acupoints (for example, skills on identifying landmarks and the Deqi sensation) and acupressure techniques (for example, skills on sustaining the pressure by fingers). The test was performed on both the elderly volunteers and the trainer (that is, one of the six members of the TCM expert panel). The interventionist could only pass the test when the skills were observed by the trainer to be up to standard and the pressure felt by both the elderly volunteer and the trainer to be optimal (that is, experience the Deqi sensation sustainably for 3 minutes) as depicted in the protocol. To prevent fatigue, one interventionist was assigned to conduct acupressure for less than 10 sessions in a whole day and adequate rest time between sessions was provided. All data collectors and research assistants have to pass the skill examination stipulated by the researchers before they can become involved in this study. The training content for the personnel conducting the intervention strictly followed the intervention protocol of the study, with complete agreement by the expert panel. The training content for data collection made reference to the original instruction manual of the related instruments [34, 41].

In order to control the quality of the intervention and data collection, all the data collectors and those conducting the intervention have to attend a separate skill evaluation provided to the research team monthly. There are also on-site visits once to each site to monitor the skill of the data collectors and those conducting the intervention. Dropout, withdrawal, undelivered intervention and uncollected data are recorded until completion of the study.

### Safety and adverse events

All adverse events (for example, unfavorable or unexpected signs and symptoms after the treatment) are monitored. Regular meetings are held among the research team and the RCH staff in order to review the trial protocol and participant safety, and to explore possible adverse events. Participants who experience suspected adverse events are excluded from the study after discussion among the research team, RCH staff and the family. If there is clear evidence of any threat to the safety of the participant, the trial will be terminated.

### Ethics review and informed consent

The study protocol was reviewed and approved by the Human Subjects Ethics Sub-committee of The Hong Kong Polytechnic University (HSEARS20120920001). In the recruitment phase, before the randomization, all the next-of-kin of subjects screened to be eligible are informed of the details of the study, including intervention and data collection procedures, potential risk and benefits. All eligible subjects are likewise informed of the same details. The subjects and the next-of-kin are clearly told about the equal chance of allocation to any one of the three groups before signing the informed consent. Subjects are recruited when 1) they sign the written informed consent (if they are sufficiently cognitively sound) or assent to participate (that is, nodding, verbalizing, or not rejecting when acupressure is performed on them), and 2) the next-of-kin sign the written proxy consent [42]. Due to the principle of blinding, only the participants allocated to the usual-care group will be told to wait for their free compensatory acupressure treatments after completion of the study. The group allocation status will be strictly concealed to the participants, next-of-kin, data collectors, and nursing home staff throughout the study period. The group allocation will only be revealed after completion of the study.

### Statistical methods

#### Sample size estimation

To estimate the sample size using G*Power® [43], with assumptions (that is, α =0.05, power =0.8, three groups), estimated effect size (that is, f =0.27) and attrition rate (that is, 10%) from the pilot study [32] and the previous similar studies [26, 27], the total number of samples needed in a randomized controlled trial to demonstrate a significant mean difference among the three groups is 99. There will therefore be 33 participants in each group.

#### Analysis

Data analysis will be performed by a statistician blinded to the whole trial process using IBM SPSS Statistics 20 software [44]. The level of significance will be reported at *P* <0.05. Both per-protocol and intention-to-treat analysis will be used in order to provide more information on the effect of the intervention, considering possible confounders such as non-compliance, protocol deviations and withdrawal [45]. The per-protocol population is defined as participants who completed the study without major protocol violation (for example, who received more than 80% of the acupressure sessions). The intention-to-treat population is defined as participants who are randomized into groups after the collection of baseline data. Demographic and clinical data will be analyzed at baseline to measure the balance among the three groups. The results will be described by mean and standard deviation for continuous data, and by frequency and percentage for categorical data. The general estimated equation model will be used to compare the effects among groups and time points.

## Discussion

The results of this study will primarily answer the question whether acupressure demonstrates a significant effect in a tightly controlled trial (with maximum possible blinding and stringent control of the intervention implementation fidelity). One of the key active components of acupressure, differentiating it from other manipulative therapies (for example, massage, therapeutic touch), is the activation of acupoints. This study first provides a sham-acupressure group to compare the efficacy difference of acupressure between tactile stimulation and acupoint stimulation. This study uses a relatively shorter intervention duration. A shorter duration of therapy may favor greater compliance with the intervention in the clinical application. This study is the first to use a bio-marker (that is, salivary cortisol) and the extent of the use of psychotropic drugs to track the outcome of the intervention. These may provide a more objective comparison of effects. Finally, this study is also the first to examine the delayed effect of the intervention. This may also provide a reference for clinical application.

## Trial status

The trial is currently recruiting participants.

## Abbreviations

AG: acupressure group
CMAI: Cohen-Mansfield Agitation Inventory
PWD: people with dementia
RCH: residential care home
TCM: traditional Chinese medicine.
